# Reliability and validity of the Healthy Home Survey: A tool to measure factors within homes hypothesized to relate to overweight in children

**DOI:** 10.1186/1479-5868-5-23

**Published:** 2008-04-28

**Authors:** Maria J Bryant, Dianne S Ward, Derek Hales, Amber Vaughn, Rachel G Tabak, June Stevens

**Affiliations:** 1 Clinical Research Unit, University of Leeds, Leeds, LS2 9NG, UK; 2 Department of Nutrition, School of Public Health, University of North Carolina, North Carolina, 27599, USA

## Abstract

**Background:**

The contribution of the environment to the obesity epidemic is well recognized. Parents have control over their home environment and can, therefore, support healthy dietary and activity habits in their children by manipulating factors such as access to energy-dense foods, availability of physical activity equipment, and restricting screen time. This paper describes the development of the Healthy Home Survey and its reliability and validity. The Healthy Home Survey was designed to assess characteristics of the home environment that are hypothesized to influence healthy weight behaviors in children including diet and physical activity.

**Methods:**

We recruited 85 families with at least one child between 3–8 years. The Healthy Home Survey was administered to parents via telephone and repeated in a random sample of 45 families after 7 days. In-home observations were performed within 14 days of the first Healthy Home Survey interview. Percent agreement, Kappa statistics, Intra-class correlation coefficients and sensitivity analyses were used to evaluate reliability and validity evidence.

**Results:**

Reliability and validity estimates for the Healthy Home Survey were varied, but generally high (0.22–1.00 and 0.07–0.96 respectively), with lower scores noted for perishable foods and policy items. Lower scores were likely related to actual change in the perishable foods present and the subjective nature or clarity of policy questions and response categories.

**Conclusion:**

Initial testing demonstrated that the Healthy Home Survey is a feasible, reliable, and valid assessment of the home environment; however, it has also highlighted areas that need improvement. The Healthy Home Survey will be useful in future research exploring the relationship between the home environment and child weight.

## Background

Worldwide, it is estimated that 10% of school-age children are currently overweight or obese with rates continuing to rise[1]. In the US, the most recent data suggest that 34% of children are overweight (>85^th ^percentile) and 17% are obese (>95^th ^percentile)[2]. Given these alarming trends, increased attention has been focused on understanding the etiology of obesity and possible management strategies, particularly the contribution of the environment [1,3-5]. The current environment has been described as "toxic" or "obesogenic'" due to the presence of an almost unlimited, convenient supply of highly palatable, energy dense foods, coupled with conditions that encourage sedentary behaviors and discourage physical activity [1,4,6]. Most environmental research to date has focused on the impact of macro-level environments such as neighborhoods and communities on healthy weight behaviors. Such research has launched a wave of 'active living' initiatives that promote building, neighborhood and road network designs that encourage communities to become more active [7-9].

Micro-level, or 'home' environments are also likely to correlate to overweight in children, but there has been less attention in this area of research [1,6,10]. Physical and social parameters of the home environment hypothesized to influence children's diets include the foods available in the home, parents' own eating habits, and feeding practices parents employ [11-14]. For example, the availability and accessibility of foods in the home may be a major factor influence on childhood diet, since children's food intake is largely dependent upon provision of food by others [15,16]. Studies in this area have found that an increase in the availability of foods is related to the consumption of those foods [16-19]. Many studies have considered fruits and vegetables specifically, finding that when fruits and vegetables were available, children were more likely to eat them.

In addition to making healthy foods available and accessible in the home, most researchers would agree that parents can encourage their children to eat more healthfully by adopting appropriate feeding behaviors and practices. Research has shown that what parents eat themselves (i.e., parental modeling) has a strong influence on children's food preferences and intake [11,20-23]. Also, parental restriction has been positively associated with overeating, or eating in the absence of hunger, especially in girls [24]. The few studies examining parental disinhibition [25] and neglect [26] suggest that both may lead to poorer dietary habits in children. Family meals are also likely to influence diet in children with evidence showing that skipping breakfast (or eating breakfast less often) is related to increased body mass index (BMI) in children [27-30] and eating family dinners is associated with more healthful dietary intake patterns, including greater consumption of fruits and vegetables [31].

Access to and provision of environments which encourage or discourage physical activity are also likely to influence childhood body composition, although this area has received somewhat less attention compared to food and eating behaviors. As with diet, a child's activity may be influenced by both social and physical parameters in the home, such as parents' own physical activity habits, parents' rules and policies around play, encouragement to play, restriction of sedentary activities and provision of areas for active play. Environmental determinants of children's physical activity have been examined in a recent review by Ferreira et al[32]. Parental physical activity (i.e. role modeling) has been studied extensively, but with mixed results. Father's activity was most consistently associated with child physical activity. Time spent outdoors, although less studied, was an area consistently associated with higher activity levels in children. Another major area of focus has been the impact of TV watching, with many studies finding positive correlations between the amount of time spent watching TV and BMI [33]. Discrepancies in findings regarding the TV-childhood obesity relationship may be due to low quality measurements of TV exposure [34,35]. Given the challenges in measuring the amount of time spent watching TV, assessment of factors such as the number and location of TVs and parental restriction of access to TVs should be explored as possible predictors of sedentary behaviors.

Interventions that focus on improving the physical and social environments in the homes of children need to be tested. One impediment to this research is the lack of tools with sound reliability and validity evidence that can be used to measure factors within the home that may relate to healthy weight behaviors in children. The Healthy Home Survey (HHS) was developed to address this measurement gap. The purpose of this paper is to describe the development of the HHS, as well as the reliability and validity testing of this instrument.

## Methods

### Survey development

Development of the Healthy Home Survey began with a review of the literature for confirmed or hypothesized associations between physical and social characteristics of the home environment and healthy weight in children. An unrestricted list of potential questionnaire items was generated to measure characteristics of the home environment related to food, physical activity and media. Specific domains included food availability (presence/absence, variety and quantity), eating environment and policies, physical activity environment, physical activity policies, media environment and media policies.

The list of potential questionnaire items was circulated to 5 experts in the field. Feedback was requested with regard to the relevance of the items and the factors of interest, clarity of wording, and identification of items which should be added or removed. After incorporating this feedback, the questionnaire was pre-tested in a sample of five parents of children ages 3–8 years to ensure that representatives of the study sample understood the meaning of each item, as well as the clarity of the wording and response options. Amendments were made as needed [Additional file 1].

Since this was the first phase of the HHS development, a number of open response items were included in order to capture all potentially important responses. For example, food availability questions asked the participant to describe all foods that they had in their homes within each food category; fruit (fresh, dried, frozen, canned/jarred), vegetables (fresh, frozen, canned/jarred), sweet snacks (e.g. cookies, ice-cream, Twinkies, muffins, cake), salty snacks (e.g. peanuts, chips, tortillas, pretzels), candy (hard candy, chocolate bars) and soda (not diet). Other response options were chosen to accommodate characteristics of each item. Eating practices, for example, queried number of days the child ate breakfast at home; thus, the response was the number of days (0–7). More subjective items, such as "do you reward your child with desserts" were given Likert-type scale response options (e.g., all of the time, most of the time, some of the time, rarely or never). A copy of the questionnaire is available from the first author by request.

### Sample

We recruited 85 families with at least one child between the ages of 3–8 years using newspaper advertisements, list-serves and community postings. Inclusion criteria were: having at least one child between ages 3 and 8 years old living in the home; residing within 20 miles of the University of North Carolina (UNC) at Chapel Hill; having lived in residence at least 6 months with no plans to move residences within the next 3 months; and agreement to participate in the home visits and assessments. If there was more than one eligible child in a family, the eldest child was allocated to be the study reference child. We certify that all applicable institutional and governmental regulations concerning the ethical use of human volunteers were followed during this research. All study procedures were reviewed and approved by the UNC Institutional Review Board.

### Procedures

A combination of telephone interviews and home visits were used to produce reliability and validity evidence for the HHS. The survey was administered by telephone to the parent that originally responded to advertisements while in their home. Start and stop times were recorded in order to calculate duration of the interview and assess feasibility. Approximately 50% of participants (n = 45) were randomly selected to receive a second telephone interview to evaluate test re-test reliability of the measure. Participants were assigned to receive the second interview using alternating allocation as they responded to recruitment advertisements. The second telephone call was planned for 7 days (+/- 1 day) after the first call.

Approximately 7 days (but no more than 14 days) after the initial telephone interview, a home visit was planned so that researchers could objectively measure the physical items. Height, weight, physical activity, and diet data were collected for the reference child and parent in order to examine possible associations between these variables and characteristics of the home environment; however, these results are not presented in this paper.

Data was collected by a team of 3 researchers, following training for the collection of both the telephone interview and home visit data. Two staff performed each home visit and any subjective decisions (e.g. 'usability of the dinning room table' or 'adequate play space') were determined through consensus of both researchers. Staff were continuously monitored and supervised throughout data collection to ensure quality control.

Participants were asked to report all food and drink items within each food category. Researchers then confirmed their relevance to each category based on pre-determined lists of foods and drinks, training and consensus. Prompts were provided to aid completeness (e.g. "what about raisins in your baking cupboard" when enquiring about dried fruit).

### Statistical Analysis

Food availability variables (i.e., 'variety' and 'quantity') were derived from the data. Variety refers to the number of different types of foods within each category. If the participant responded "yes" to the presence of fruit in their home, they would be asked to describe the fruits including an estimation of the quantity. For example, if a participant reported having 2 apples, 2 bananas and 1 orange, the variety score for fruit would equal 3. Quantity was derived by looking at typical food and package sizes (e.g. small, medium, and large). Local grocery store and manufacturer websites were used to determine the range of size options available in order to set a standard size (quantity) for each food item in the database. For example, we found that boxed raisins were typically available in small and large size options. Based on the median weight for each of these sizes available from different manufactures, standard sizes for boxes of raisins were set at 1.5 oz for small and 13.5 oz for large. The Nutrition Epidemiology Core at UNC Clinical Nutrition Resource Center (DK056350) used the University of Minnesota Nutrition Data System for Research to calculate the number of servings of food available.

Statistical analyses were performed using SAS (v9.1, SAS Institute Inc, Cary, NC, 2003). Percent agreement, Kappa statistics [36,37] proportion of positive and negative agreement [38,39] sensitivity, specificity and single measure Intraclass correlation coefficients (ICC) [40] were used to evaluate reliability and validity evidence. For items with more than two ordered response options, weighted kappa was estimated. As a guide, we followed the benchmarks suggested by Landis and Koch [15] for agreement: < 0.00 = poor, 0.00 – 0.20 = slight, 0.21 – 0.40 = fair, 0.41 – 0.60 = moderate, 0.61 – 0.80 = substantial and 0.81 – 1.0 = almost perfect. Response prevalence for several items was skewed; therefore the prevalence and bias adjusted Kappa (PABAK) [38] was calculated for any item where 85% or more of the sample responded in one direction. The proportion of positive agreement is the number of "yes" agreements (person responds "yes" at both phone 1 and phone 2) divided by the average number of "yes" responses from the two phone interviews. The proportion of negative agreement is the number "no" agreements (person responds "no" at both phone 1 and phone 2) divided by the average number of "no" responses from the two phone interviews. These separate indexes allow for distinction in item performance that a single estimate (e.g. kappa or PABAK) may obscure. For example, agreement may be high for an item when a person responds "yes" (Ppos = 75%), but if someone says "no" on one occasion they may be unlikely to respond "no" during another interview (Pneg = 50%). This may be important if trying to identify a specific group or behavior. For sensitivity and specificity, the home visit was used as the criterion measure. Sensitivity is the proportion of people reporting "yes" during phone interview 1 that were confirmed as 'yes' during the home visit. Specificity is the proportion of people reporting "no" during phone interview 1 that were confirmed as 'no' during the home visit. All ICCs are single measure estimates and were calculated using the INTRACC macro developed for SAS by Hamer [41].

## Results

### Participant characteristics

The mean age of children and parents was 5.05 (S.D. 1.34) and 35.81 (S.D. 5.99) years respectively. There were slightly more boys (56%) than girls and more female primary caregivers (98%) compared to males. For children, mean body mass index (BMI- weight (kg)/height^2 ^(m)) percentile was 64% (28.01). For primary care givers, average BMI was 26.81 (S.D. 6.70). Most participants were White (72.9%) or African American (23.5%) and lived in detached homes (83.7%). A range of household incomes were reported with 10.5% reporting less than $19,000, 19.8% reporting $20,000–$49,000; 50.0% reporting $50,000–$100,000 and 12.8% reporting greater than $100,000. Seven percent of participants declined to report annual household income (Data not shown).

### Feasibility

All 85 families completed the initial telephone interview. The mean duration of these interviews was 34.4 (SD 9.2) minutes. All 45 families asked to complete a second telephone interview did so. The mean duration of the second interview was 29.0 (SD 8.6) minutes and, on average, they took place 7.6 days (SD 1.3) after the initial telephone interview. More than 95% of intended home visits (81/85) were completed. Four home visits could not be completed due to scheduling problems that prevented the completion of visits within the predetermined time frame. The mean number of days between the first interview and the home visit was 7.9 (SD 3.6) days (in line with the prescribed protocol of 7–14 days).

### Food availability: presence/absence

Table 1 shows the test re-test reliability and validity of food availability items (yes/no). For reliability, percent agreement was almost perfect for all items (ranging between 84.44% – 95.56%). Kappa scores were more variable, ranging from -0.02 to 0.86. Responses to many of the items with lower kappa values were skewed, with 85% or more of the sample reporting they were available in the home. Prevalence and bias adjusted Kappas (PABAK) were greater than 0.75 for each of these items. The proportion of positive and negative agreement ranged from 0.91 to 0.98 and 0.00 to 0.91 respectively.

**Table 1:** Food Availability: Yes/No

	**Reliability (n = 45)**					**Validity (n = 82)**				
		
	**%Agree**	**Kappa (95% CI)**	**PABAK**^1^	**Ppos**	**Pneg**	**%Agree**	**Kappa (95% CI)**	**PABAK**^1^	**SEN**	**SPEC**
Fresh fruit	93.2	0.00 (-0.00–0.00)	0.86	0.97	0.00	93.9	-0.02 (-0.05–0.01)	0.88	0.95	0.00
Canned fruit	86. 7	0.59 (0.29–0.88)	0.83	0.92	0.67	86.6	0.48 (0.22–0.74)	0.73	0.93	0.58
Frozen fruit	93.3	0.86 (0.70–1.00)	n/a	0.95	0.91	93.9	0.87 (0.77–0.98)	n/a	0.92	0.97
Dried fruit^2^	91.1	0.74 (0.51–0.98)	n/a	0.94	0.80	93.9	0.85 (0.72–0.98)	n/a	0.93	0.86
Fresh vegetables	93.3	0.63 (0.25–1.00)	0.86	0.96	0.67	92.7	0.23 (-0.15–0.61)	0.85	0.93	1.00
Canned vegetables	95.6	0.78 (0.48–1.00)	0.91	0.96	0.67	93.9	0.67 (0.40–0.94)	0.83	0.95	0.50
Frozen vegetables	95.6	0.48 (-0.14–1.00)	0.87	0.97	0.00	91.5	0.42 (0.07–0.77)	0.85	0.95	0.60
Salty snacks	95.6	-0.02 (-0.05–0.01)	0.91	0.98	0.00	96.3	0.00 (-0.00–0.00)	0.93	0.96	
Sweet snacks	95.6	0.64 (0.18–1.00)	0.91	0.98	0.67	93.9	0.00 (-0.00–0.00)	0.88	0.94	
Candy^2^	88.6	0.48 (0.09–0.87)	0.77	0.94	0.55	86.6	0.45 (0.18–0.72)	0.73	0.89	0.67
Soda	84.4	0.63 (0.38–0.88)	n/a	0.91	0.77	80.5	0.54 (0.34–0.74)	n/a	0.83	0.73

There was greater than 80% agreement between the phone and home visit for all food categories. Kappa scores varied ranging from -0.02 to 0.87. PABAK was 0.73 or greater where presented. Sensitivity was generally high (range 0.83 to 0.96), while specificity ranged from 0.00 to 1.00).

### Food availability: Variety and number of servings

Similar to presence or absence of food availability, test re-test reliability for food variety was high (substantial to almost perfect) for most items (Table 2). One exception was fresh fruit, with an ICC of only 0.37 (95% CI = 0.09–0.59). Validity for the variety of food items was similar to reliability (ICC ranging between 0.30 – 0.82), with a lower ICC also observed for fresh fruit. In addition, results for sweet snacks were considered as only fair (ICC = 0.30; 95% CI = 0.09–0.49).

**Table 2:** Single measure Intraclass correlation coefficients and 95% confidence intervals for food variety and servings

	**Variety (number of types)^1^**		**Servings (number of servings)**	
		
	**Reliability (95% CI)**	**Validity (95% CI)**	**Reliability (95% CI)**	**Validity (95% CI)**
Fresh fruit	0.37 (0.09–0.59)	0.54 (0.37–0.68)	0.22 (-0.07 – 0.48)	0.39 (0.20–0.56)
Canned fruit	0.81 (0.68–0.89)	0.66 (0.51–0.76)	0.84 (0.72 – 0.91)	0.68 (0.55–0.78)
Frozen fruit	0.85 (0.75–0.92)	0.71 (0.59–0.81)	0.91 (0.84 – 0.95)	0.83 (0.76–0.89)
Dried fruit	0.89 (0.81–0.94)	0.88 (0.82–0.92)	0.39 (0.11 – 0.61)	0.67 (0.53–0.77)
Fresh vegetables	0.81 (0.70–0.90)	0.70 (0.57–0.80)	0.77 (0.62 – 0.87)	0.30 (0.09–0.48)
Canned vegetables	0.89 (0.81–0.94)	0.70 (0.57–0.79)	0.81 (0.68 – 0.89)	0.68 (0.54–0.78)
Frozen vegetables	0.83 (0.71–0.90)	0.82 (0.73–0.88)	0.63 (0.42 – 0.78)	0.74 (0.63–0.83)
Salty/savory snacks	0.85 (0.75–0.92)	0.48 (0.29–0.63)	0.71 (0.53 – 0.83)	0.46 (0.28–0.61)
Sweet snacks	0.65 (0.44–0.77)	0.30 (0.09–0.49)	0.64 (0.42 – 0.78)	0.32 (0.12–0.50)
Candy	-^2^	-^2^	0.49 (0.23 – 0.68)	0.64 (0.50–0.75)
Soda	0.77 (0.62–0.87)	0.76 (0.65–0.85)	0.69 (0.50 – 0.82)	0.66 (0.53–0.77)

^1^Calculated from average serving size for each item; ^2^Variety of candy not calculated

Reliability results for the number of servings of each item are also shown in Table 2. ICCs ranged from 0.22–0.91 and were lowest for fresh (ICC 0.22, 95% CI = 0.07–0.48) and dried fruit (ICC 0.39, 95% CI = 0.11–0.61). Validity estimates for servings were lowest for sweet snacks (ICC 0.32, 95% CI = 0.12–0.50) and fresh vegetables (ICC 0.30, 95% CI = 0.09–0.48), and highest for frozen fruit (ICC 0.83, 95% CI 0.76–0.89) and frozen vegetables (ICC 0.74, 95% CI = 0.63–0.83).

### Food environment

Reliability and validity estimates for food environment items are shown in Table 3. Percent agreement for reliability (mean = 86%) and validity (mean = 71%) ranged from 58% to 98%. Agreement across phone interviews was generally good with most Kappa estimates above 0.60, with the lowest scores found for fresh fruit (κ = 0.49, 95% CI = 0.19–0.79). Kappa estimates for validity were low (all but one less than 0.50). Sensitivity was, however, greater than 0.75 for four items.

**Table 3:** Reliability and validity estimates for Food environment items from the HHS

	**Reliability**						**Validity**					
		
	**n**	**%Agree**	**Kappa (95% CI)**	**PABAK^1^**	**Ppos**	**Pneg**	**n**	**%Agree**	**Kappa (95% CI)**	**PABAK^a^**	**SEN**	**SPEC**
Fruit in view	44	81.8	0.49 (0.19–0.79)		0.88	0.60	78	78.2	0.32 (0.06–0.57)		0.89	0.41
Vegetables ready to eat	43	79.1	0.57 (0.32–0.82)		0.82	0.74	74	75.7	0.43 (0.22–0.65)		0.79	0.67
TV in view of dining area	45	91.1	0.82 (0.65–0.99)		0.92	0.90	78	78.2	0.57 (0.38–0.75)		0.82	0.75
Adequate counter space	45	97.8	0.88 (0.64–1.00)	0.96	0.99	0.89	78	92.3	0.22 (-0.18–0.62)	0.85	0.99	0.17
Access to candy	43	86.0	0.72 (0.52–0.93)		0.87	0.83	73	60.3	0.22 (0.00–0.43)		0.57	0.68
Access to soda	41	85.4	0.71 (0.49–0.92)		0.83	0.82	65	61.5	0.26 (0.06–0.47)		0.54	0.77
Access to sweet snacks	44	81.8	0.62 (0.39–0.85)		0.77	0.85	78	65.4	0.29 (0.10–0.49)		0.63	0.73
Access to savory snacks	44	84.1	0.68 (0.47–0.90)		0.84	0.84	78	57.7	0.07 (-0.15–0.29)		0.63	0.45

^1 ^Adjusted Kappa (PABAK) used if prevalence ≥85%; Ppos = proportion of positive agreement; Pneg = proportion of negative agreement; SEN = sensitivity (proportion of those reporting YES confirmed as YES by the home visit); SPE = specificity (proportion of those reporting NO confirmed as NO by the home visit); CI = confidence interval

### Eating practices and eating, media and physical activity policies

Table 4 shows the reliability scores for policies and practices within the home related to eating, media and physical activity. It was not possible to assess criterion validity, since these items are not physically observable. For eating practices, there was generally good agreement between telephone responses with ICCs ranging from 0.64 to 0.92. The lowest, but still moderate, kappa and ICC values were noted for items asking parents to estimate the number of days per week the child eats lunch watching TV and eats snacks watching TV.

**Table 4:** Eating practices and eating, media and physical activity policies (reliability only)

**N = 45**	**% Agree**	**Kappa (95% CI)**	**ICC (95% CI)**
	
**Eating practices (days/wk)**			
Breakfast at home	88.9	0.86 (0.74–0.98)^w^	0.93 (0.87–0.96)
Breakfast at school	77.8	0.81 (0.70–0.91)^w^	0.91 (0.85–0.95)
Breakfast elsewhere	86.7	0.64 (0.42–0.87)^w^	0.71 (0.53–0.83)
Breakfast watching TV	64.4	0.78 (0.66–0.90)^w^	0.89 (0.80–0.94)
Lunch watching TV	77.8	0.52 (0.29–0.76)^w^	0.66 (0.46–0.80)
Dinner watching TV	73.3	0.80 (0.70–0.90)^w^	0.92 (0.86–0.96)
Snacks watching TV	60.0	0.62 (0.44–0.80)^w^	0.69 (0.51–0.82)
Dinner away from home^1^	65.9	0.59 (0.43–0.74)^w^	0.64 (0.42–0.79)
Dining together	75.6	0.79 (0.66–0.92)^w^	0.89 (0.81–0.94)
**Eating policies (categorical)**			
Location eat dinner	93.3	0.73 (0.46–0.99)	-
Amount served to child	95.6	0.57 (0.13–1.00)^w^	-
Finish dinner policy ^2^	75.0	0.75 (0.51–0.91)^w^	0.79 (0.65–0.88)
Restriction of desserts	53.3	0.61 (0.46–0.77)^w^	0.75 (0.60–0.86)
Reward with desserts	55.6	0.58 (0.43–0.73)^w^	0.71 (0.53–0.83)
Seconds policy	64.4	0.36 (0.11–0.61)^w^	0.32 (0.04–0.56)
Set meal times policy	42.2	0.40 (0.20–0.60)^w^	0.52 (0.27–0.70)
Child serves own dinner^2^	63.6	0.68 (0.53–0.84)^w^	0.77 (0.61–0.87)
Child serves own snacks	68.9	0.65 (0.49–0.82)^w^	0.77 (0.62–0.87)
Parent avoids snacking	57.8	0.61 (0.47–0.76)^w^	0.76 (0.61–0.86)
Parent eats healthy	66.7	0.44 (0.21–0.67)^w^	0.44 (0.17–0.65)
**Media and physical activity policies (categorical)**			
Restrict TV use	71.1	0.68 (0.54–0.83)^w^	0.79 (0.65–0.88)
Restrict Computer use	60.0	0.62 (0.44–0.80)^w^	0.66 (0.47–0.80)
Restrict Video Game use^3^	66.7	0.58 (0.35–0.81)^w^	0.60 (0.35–0.77)
Reward with TV	77.8	0.77 (0.63–0.92)^w^	0.82 (0.70–0.90)
Reward with Computer	82.2	0.74 (0.59–0.88)^w^	0.84 (0.72–0.91)
Reward with Video Game^3^	87.2	0.75 (0.50–1.00)^w^	0.86 (0.74–0.93)
Restrict Active indoor play	53.3	0.49 (0.30–0.67)^w^	0.68 (0.48–0.81)
Restrict outdoor play (yard)^4^	51.2	0.41 (0.21–0.61)^w^	0.54 (0.29–0.72)
Restrict outdoor play (neighborhood)	53.3	0.52 (0.34–0.71)^w^	0.63 (0.41–0.78)

^w^Weighted Kappa (items with ≥3 response categories); ICC = single measure intraclass correlation coefficient; ^1^n = 41; ^2^n = 44; ^3^n = 39; ^4^n = 43

Kappa scores for eating policies varied (range 0.36–0.75), although most were considered at least moderate. Three items were noted as having substantially lower percent agreement, Kappa, and/or ICC estimates. Whether a child was only permitted to eat at set meal times had the lowest percent agreement (42.22%) while policy of having second helpings (ICC 0.32, 95% CI = 0.04–0.56) and whether a parent considers themselves to eat healthy (ICC 0.44, 95% CI = 0.17–0.65) had questionable ICC and kappa values.

Kappa and ICC estimates for media and physical activity policy items ranged from 0.41 to 0.86. The highest reliability estimates were noted for the three "reward" media items. The lowest Kappa and ICC were for restriction of outdoor play in the yard (κ = 0.41; ICC = 0.54).

### Physical activity and media environment

Reliability and validity results for physical activity and media environment items are shown in Table 5. The mean kappa value for the physical activity and media environment reliability was 0.81. The presence of a bike or riding toy was found to have a low Kappa (κ = 0.29, 95% CI = -21–0.79), but high percent agreement (91%) due to the fact that 98% of parents reported the presence of a bike or riding toy. Similar results were observed for the validation of the bike item. A low kappa (κ = 0.06, 95% CI = -0.11–0.22) was also noted for 'adequate play space inside' suggesting discordance between observers and parental rating. Validity estimates for yard size (κ = 0.49, 95% CI = -0.31–0.66) and computer in child's bedroom (κ = 0.53, 95% CI = -0.21–0.86) while acceptable, were lower than expected.

**Table 5:** Reliability and validity estimates for physical activity and media environment items from the HHS

	**Reliability**						**Validity**					
	**n**	**% Agree**	**Kappa (95% CI)**	**PABAK**^1^	**Ppos**	**Pneg**	**n**	**% Agree**	**Kappa (95% CI)**	**PABAK**^1^	**SEN**	**SPEC**
**Physical activity environment**												
Outdoor recreation facilities	45	91.1	0.66 (0.36–0.97)		0.95	0.71						
Indoor recreation centers	45	95.6	0.88 (0.72–1.00)		0.91	0.97						
Parent exercise (past month)	45	95.6	0.73 (0.36–1.00)		0.98	0.75						
Yard	45	100.0	1.00	1.00	1.00	1.00	79	98.7	0.85 (0.56–1.00)	0.98	1.00	0.75
Yard Size	43	83.7	0.73 (0.54–0.92)^w^				75	66.7	0.49 (0.31–0.66)^w^			
Share Yard	43	97.7	0.85 (0.55–1.00)	0.95	0.86	0.99	75	96.0	0.71 (0.40–1.00)	0.92	1.00	0.96
Play equipment in Yard	43	95.4	0.90 (0.78–1.00)		0.94	0.96	74	90.5	0.81 (0.67–0.94)		0.98	0.82
Bike or riding toy	45	91.1	0.29 (-0.21–0.79)	0.82	0.95	0.33	78	96.2	-0.02 (-0.04–0.01)	0.92	0.99	0.00
Adequate Play Space Inside	45	77.8	0.69 (0.50–0.89)^w^				79	43.0	0.06 (-0.11–0.22)^w^			
**Media environment**												
Number of TVs	45	100.0	1.00^w^				78	84.6	0.88 (0.82–0.95)^w^			
Number of DVD players	45	75.6	0.77 (0.64–0.90)^w^				79	59.5	0.56 (0.43–0.70)^w^			
Number of computers	45	93.3	0.92 (0.84–1.00)^w^				79	64.6	0.58 (0.45–0.72)^w^			
Number of video games consoles	45	88.9	0.84 (0.73–0.95)^w^				79	73.4	0.64 (0.55–0.74)^w^			
Number of DVDs	44	75.0	0.56 (0.33–0.80)^w^				79	64.6	0.60 (0.46–0.75)^w^			
Cable TV	45	100.0	1.00		1.00	1.00	79	98.7	0.96 (0.89–1.00)		1.00	0.94
TV in child's bedroom	45	100.0	1.00		1.00	1.00	79	96.2	0.88 (0.75–1.00)		0.88	0.99
Computer in child's bedroom	45	100.0	1.00	1.00	1.00	1.00	79	92.4	0.53 (0.21–0.86)	0.85	0.44	0.99
Video game in child's bedroom	42	97.6	0.84 (0.55–1.00)	0.95	0.86	0.99	76	94.7	0.72 (0.47–0.98)	0.90	0.60	1.00

^w^Weighted Kappa (for items with ≥3 response categories); ^1 ^Adjusted Kappa (PABAK) used if prevalence ≥85%; Ppos = proportion of positive agreement; Pneg = proportion of negative agreement; SEN = sensitivity (proportion of those reporting YES confirmed as YES by the home visit); SPE = specificity (proportion of those reporting NO confirmed as NO by the home visit); CI = confidence interval

## Discussion

To our knowledge, this study was the first to develop and evaluate a tool which measures both physical and social factors within the home setting that influence diet, physical activity, and sedentary behaviors of children. The instrument was found to be feasible and the majority of items demonstrated substantial to almost perfect agreement between the two phone interviews and between the first phone interview and the home assessment.

Assessment of the home environment did have some challenges. One area in particular that needs to be revisited is the assessment of food availability in the home, especially the quantity or number of servings available. Calculating servings required a number of assumptions. As described previously, package sizes were captured as small, medium, or large. In order to calculate the number of servings, these general sizes had to be translated into more exact volume or weight quantities. This methodology likely affected the reliability and validity of these items. Although the HHS was not intended to provide a complete food inventory, it may be valuable to capture more detailed package size information in future work with this instrument.

Although the majority of items in the survey had moderate to high reliability, somewhat lower scores were noted for variety and quantity of fresh fruit, 'fruit in view', 'seconds policy', 'set meal times', 'parent eats healthy', and 'restrict outdoor play'. Low reliability scores for variety and quantity of fresh fruit (and possibly fruit in view) may reflect actual changes due to consumption or purchase during those 7 days between the first and second telephone interviews, since fresh fruits usually perish within this time frame. While current data do not allow separation of the variance attributed to natural change, it will be important to determine so that data collection methods and questionnaire items could be modified to improve the quality of data collected. We believe that measurement of the variety and quantity of foods may be a better indicator of the quality of foods in the home compared to if presence or absence alone is measured. For example, a dictomous measure could report that fruit is available in a home when just one apple is present. On the other hand, a measure of the quantity of different fruits would distinguish whether a home contained just one apple or a variety of different fruits. For the purpose of this tool evaluation study, we did not take into account the variables which are likely to influence quantity or quality, including the number of days since shopping, or the number of people living in the home. Such factors would be considered when using the measure as a means to describe a family's home environment.

Low reliability for the three questions about eating and media policies may be the result of confusion about the meaning of these questions. For example, data collectors noted that a number of participants needed additional clarification about the 'seconds policy' question before being able to answer, suggesting that some questions need to be revised. Other items with lower scores for reliability were of a more subjective nature (e.g. parent self-report of whether they eat healthy).

In general, moderate to high validity was observed; although slightly lower scores were noted for some items on variety and the food environment. The variety items with the lowest validity were salty and sweet snacks and fresh fruit. Lower validity for items measuring snack variety may have been a function of how individual participants versus trained data collectors reported similar items either together or separately. For example, if a participant had 1 bag of potato chips and 1 bag of tortilla chips in their pantry, they may have reported "2 bags of chips." Trained data collectors would have distinguished between the two and recorded 1 bag of each. As a result, calculated variety based on participant report and direct observation would be different. Food environment items that demonstrated lower validity included 'fruit in view', 'adequate play space inside' and items ascertaining the degree of accessibility of candy, soda, and snacks. The subjective nature of these items likely impacted validity estimates. For example, when interpreting what is meant by 'accessible', data collectors considered foods to accessible if children could reach the item without assistance, including if there was a chair nearby to help them reach higher items. It is possible that parents' did not take this into consideration or that they believed that their child did not have access because they never attempted or were not permitted to retrieve them. In an attempt to reduce such error, parents were told that accessibility was not related to permission. A further item asked the parent to report whether the child was allowed to "help themselves to snacks", which we specified was related to permission.

Quantity scores (number of servings) for vegetables were of lower validity compared to food variety. Serving sizes were based on the original package size, rather than the amount that remained in a package, except for foods that are sold as loose items (e.g. fresh vegetables). We reported package size by category (e.g. small, medium or large). Consequently, this variable was more subject to interpretation. Additionally, vegetables are perishable and an estimated bag size will change with consumption. Thus, similar to reliability, assessment of some food items may be measuring actual change rather than validity of the item scores.

An area for future work is the development of items associated with the physical activity environment, including both social and physical factors. Compared to the diet area, less is known about the role of the home environment on physical activity behavior. Future work with this instrument should identify other environmental characteristics that could be related to this behavior and to BMI levels.

Assessment of the family home environment has been popular for many years in child development research. The HOME (Home Observations for Measurement of the Environment) instrument was developed over three decades ago to measure the quantity and quality of stimulation and support available to children within their homes to facilitate optimal development. Multiple versions of this questionnaire for specific age ranges now exist, and it is used both as a measurement tool and as a means to evaluate the intervention effectiveness [42]. Recent data collected using the HOME found positive associations between items within HOME that relate to opportunity for productive activity (e.g., availability of books and games) and weight gain in children[43]. More specifically, those children who gained weight had fewer opportunities for productive activity and watched more television compared to those who did not gain weight. This study also found that children who gained weight were more likely to have parents with more controlling parenting styles. While such relationships with weight status have been observed by using the HOME tool, that instrument does not specifically target factors that influence healthy weight behaviors. More recently, Gattshall et al [44]developed and examined a survey to assess home environments in overweight children. Results demonstrated generally good reliability (physical activity items ICC = 0.43–0.96; food/diet items 0.01–0.90), but it is not possible to compare validity scores to the HHS, since Gattshall did not assess criterion validity.

Other researchers have focused more specifically on assessing the home food environment as a proxy for diet intake. This is a growing area of research, but there remains to be a lack of published manuscripts detailing the evaluation of such measures [16,21,45], and few measures exist with adequate evidence for reliability and validity of scores. Recently, Campbell et al., [22] measured predictors of childhood diet within family homes using an adolescent- and parental-report questionnaire that included assessments of food availability, child feeding practices, parental modeling and television exposure. This questionnaire was completed by a large sample and demonstrated good internal reliability; however, test re-test reliability and criterion validity were not assessed.

Measures of the home physical activity environment are even less common than those of food. Hume et al [46] recently developed and assessed an adolescent self-report questionnaire to measure perception of home and neighborhood physical activity environments. Items were both physical (e.g., presence and size of yard) and social (e.g., encouragement to be active). Test re-test reliability (9-day interval) was examined in a small sample of children and most items were found to have moderate to good agreement (physical environment ICC = 0.8–0.94; social environment 0.16–1.00). This tool may be a useful measure of youth perceptions of the physical activity environment and as a predictor of healthy weight behaviors in the home; however, the validity of the assessments is not known.

Measurement of the home media environment has received attention due to interest in the relationship between media and sedentary behaviors[47,48]. Many questionnaires have measured the duration of watching TV, and some have reasonable reliability and validity evidence. However, few of these also considered and evaluated social environments related to screen time. Salmon et. al. [48] examined the sedentary environment, including the physical media environment as well as social factors like rules and restrictions, surrounding media behaviors from both the parents' and the children's perspectives. Scores from these instruments showed moderate to good test re-test reliability, but again, validity was not assessed.

Compared to earlier questionnaires, the Healthy Home Survey takes a more holistic approach, examining a variety of physical and social factors that might influence diet, physical activity, and sedentary behaviors in children. The instrument includes many items that have not been measured or evaluated in previous studies. For some of these new items, an open response approach was needed. This approach will enable us to further develop the tool with evidence based identification of valid and discriminate items. One downside is that this approach required a lot of time for coding and analysis; however we believe that, at this stage, open response within categories added to the richness of the data. Many of the items on the HHS were subjective, and these items often had lower reliability and validity scores. These challenges highlight the need for further development of the HHS.

This study is limited in that the population was fairly homogenous with families of moderately high social economic status. It is likely that these families were more highly motivated than those that did not volunteer to take part in the study. Further testing is required in a more diverse sample. In addition, we were not able to validate items within the HHS that related to household policies (e.g., rules regarding TV exposure and dieting behaviors) since they could not be physically observed in a single observation episode. Unlike institutions such as schools, manuals or written policy guidelines that must be adhered to within family homes typically do not exist.

## Conclusion

The HHS was designed to assess multiple factors hypothesized to relate to healthy weight behaviors in children. We believe that the assessment of more than one factor is important and relevant, given the multiple factors that influence overweight in children. Although this work has provided a major step forward in the development of an instrument to assess weight-related factors in the home micro-environment, additional work is needed to improve the measurement quality of selected items and to determine usefulness in a broader range of demographic groups. More work is also needed to understand the discriminatory power and clustering of items and the potential for deletion of items to produce a shorter instrument. In addition the sensitivity of the HHS to change (e.g., intervention effects) needs to be examined, and usefulness in diverse families evaluated. Although more research is needed, the work described here has moved forward efforts to create a reliable and valid instrument to measure aspects of the home micro-environment hypothesized to contribute to obesity in children.

## Competing interests

The authors declare that they have no competing interests.

## Authors' contributions

MB, JS, DW, AV and RT made substantial contributions to conception and design of the HHS; DH led the data analysis, with assistance from RT and MB; and all authors contributed to preparation of the manuscript. All authors read and approved the final manuscript.

## Supplementary Material

Additional file 1 Healthy Home Survey operator manual. Script and procedures for administration of the HEALTHY HOME SURVEY. Click here for file
