# Supplementary material for: Reliability and validity of the Healthy Home Survey: A tool to measure factors within homes hypothesized to relate to overweight in children
Source: Int J Behav Nutr Phys Act. 2008 Apr 28;5:23. doi: 10.1186/1479-5868-5-23 (PMC2383924; doi:10.1186/1479-5868-5-23)
Supplement: Additional file 1 — Healthy Home Survey operator manual. Script and procedures for administration of the HEALTHY HOME SURVEY. [file 1479-5868-5-23-S1.doc]

**Script and procedures for administration of the HEALTHY HOME SURVEY**

The following text presents the script that should be followed during telephone interviews in which the Healthy Home Survey (HHS) is administered. Interviewers should not attempt to perform interviews with participants until they have received training and have had the opportunity to practice administration of the interview on the telephone.

*Italicized* text indicates spoken script.

All other text indicates instructions or advice.

**Making the call**

Participants have been asked to complete a form (“Availability” form) to indicate the best times they can be reached. Please read this BEFORE calling. Record all attempts that you make to contact participant, including those they were not answered and those that were inconvenient (“Participant call attempts” form). If you call at a time that was reported as convenient by the participant, but that was not suitable at that time, make a note of the time on the participant “availability” form.

Make sure that the address and directions for each participant are already entered BEFORE you call them (Q. 12 and Q. 13 below).

**The script**

Home ID:

Administered By:

Date:

Start Time:

**CALL PARTICIPANT**

*Good morning/evening. This is <your name>from <institute name>. Could I please speak to <participant name>?*

[Participant responds]

*Hello <participant name>. We have received the form that you mailed to us with consent to take part in our family home study. As we described in the consent form, the first part of the study involves a telephone interview which should take approximately 40-60 minutes. Is now a good time to conduct that interview with you?*

**If no** ….. *No problem. I will try again using another time that you suggested might be alright on your availability form. Thank you. Goodbye.*

**If yes**….. *Okay. I’ll begin with a few general questions and then move on to more specific questions about your family home environment. Please feel free to stop me at any point or ask me to clarify any questions that you don’t understand. There are no right or wrong answers. Please answer honestly. You are not being judged on any of your responses.*

**GENERAL INFORMATION QUESTIONS**

**1.** *What is your name?*

…………………………………………………………………………………………………………….....

**1.1.** *Do you have a child between the ages of 2 and 7?* Yes / No

**1.2.** *What is the name of that child?*.............................................................................................................

[refer to the child’s name throughout when <child’s name> appears]

*Please remember that when we ask you questions about “your child”, we are referring to this child only.*

**2.** *Are you the primary caregiver for <* child’s name*> that you indicated as being suitable for this study?* Yes /No

**If no**…*we need to conduct this interview with the primary caregiver. Are they available now?*

**If no…** *Ok, I will call back another time and try to get hold of that person. Thank you. Goodbye.*

**3.** *What is your relationship with* <child’s name>*?*

……………………………………………………………………………………………………………….

**4.** *How many adults older than 17 years live in your home?*

[Participant may ask if this includes people who only live in the home for some of the time (e.g. grandparents): *Only include people who live in your home all of the time.*

………………………………………………………………………………………………………………

**5.** *For each adult living in your home, beginning with you, please tell me*:

1. *What their relationship is with* <child’s name>*?* [not required for the participant]
2. *Whether they are male or female* [not required for the participant]
3. *What is your / their age?*

[Begin by asking all questions (a-c) at once and then repeat each question and get a response before moving onto the next question. Fill in responses into Subform_Adult]

Subform_Adult

| Home ID | Adult | Relationship to reference child | Gender (M/F) | Age (yr) |
| --- | --- | --- | --- | --- |
|  | 1 |  |  |  |
|  | 2 |  |  |  |
|  | 3 |  |  |  |
|  | 4 |  |  |  |

**6.** *How many children (under 18 years of age) live in your home?*

[Participant may ask if this includes children who only live in the home for some of the time (e.g. if parents are separated)]: *Only include people who live in your home all of the time.*

……………………………………………………………………………………………………………….

**7.** *For each child, beginning with* <child’s name>*, please tell me:*

1. *What their relationship is with* <child’s name>*?* [not required for the reference child]
2. *Whether they are male or female*
3. *What is their age?*

[Begin by asking all questions (d-f) at once and then repeat each question and get a response before moving onto the next question. Fill in responses into Subform_Child]

Subform_Child

| Home ID | Child | Relationship to reference child | Gender (M/F) | Age (yr) |
| --- | --- | --- | --- | --- |
|  | Reference child | N/A |  |  |
|  | 2 |  |  |  |
|  | 3 |  |  |  |
|  | 4 |  |  |  |

**8**. *From the following options, how would you describe your race? You can choose more than one?* [select response]

1. *Black or African-American*
2. *White (non-Hispanic)*
3. *Hispanic*
4. *Asian*
5. *Native Hawaiian and other pacific islander*

[Possible response maybe that the participant does not describe themselves as being any of the options]: *Ok, could you tell me which race you would describe yourself as?* [print answer above]

……………………………………………………………………………………………………………….

**9.** *How would you describe the race of* <child’s name> *(you can choose more than one)?* [select response]

1. *Black or African-American*
2. *White (non-Hispanic)*
3. *Hispanic*
4. *Asian*
5. *Native Hawaiian and other pacific islander*

[Possible response maybe that the participant does not describe their child as being any of the options]: *Ok, could you tell me which race you would describe* <child’s name> *as being?* [print answer above]

……………………………………………………………………………………………………………….

**10.** *The next question will help us organize our study results. Please do not feel obliged to answer this question if you feel uncomfortable. From the following options, please tell me which describes your annual household income?* [select response]

1. *less than $10,000*
2. *$10,000 - $19,000*
3. *$20,000 - $50,000*
4. *$50,000 - $100,000*
5. *Greater than $100,000*

……………………………………………………………………………………………………………….

**11.** *Which of the following options best describes your occupation?* [select response]

*a) Full time working outside home*

*b) P/T working outside home*

*c) Working from home for a salary*

*d) Stay at home mom (working without a salary)*

……………………………………………………………………………………………………………….

**12.** *Please can you confirm the following address as your home address* [read address entered prior to telephone call; make alternations if necessary] ………………………………………………………………………………………………………………..………………………………………………………………………………………………………………..………………………………………………………………………………………………………………..………………………………………………………………………………………………………………..………………………………………………………………………………………………………

**13.** *These are the directions we got from MapQuesting your address. Are they correct?* [read directions entered using MapQuest prior to telephone call; make alternations if necessary]

………………………………………………………………………………………………………………………………………………………………………………………………………………………………………………………………………………………………………………………………………………………………………………………………………………………………………………………………………………………………………………………………………………………………………………………………………………………………………………………………………………………………………………………………………………………………………………………………………………………………………………………………………………………………………………………………

**NEIGHBORHOOD**

**14.** *Which of the following options best describes the type of home you live in?* [select response]

*a) Apartment*

*b) Mobile home*

*c) Town house, duplex or condo*

*d) Detached home*

[A detached home is one that is not connected to any other properties, with its own boundaries]

……………………………………………………………………………………………………………….

**15.** *Would you say that your home was on a busy street with lots of traffic?*

Y/N

[Prompt the participant with examples, like *“how does it compare to other streets, like Franklin Street”*?]

……………………………………………………………………………………………………………….

**16.** *Are there parks, walking trails or outdoor recreation areas within safe walking of your home?* Yes / No / don’t know

[Possible response may be that these are within walking distance, but that they never walk there, (for any reason, like safety, time etc.). Or that, they consider it to be walking distance, but others do not (or the opposite). Record whether or not the participant believes they ARE in walking distance, even if they do not walk there themselves]

………………………………………………………………………………………………………………

**17.** *Are there in-door recreation centers that you could use within safe walking of your home (e.g. YMCA, community rec centers, school gyms)?* Yes /No / don’t know

[Possible response may be that these are within walking distance, but that they never walk there, (for any reason, like safety, time etc.). Or that, they consider it to be walking distance, but others do not (or the opposite). Record whether or not the participant believes they ARE in walking distance, even if they do not walk there themselves]

……………………………………………………………………………………………………………….

**18.** *Does the street that you live in have a side walk?* Yes /No

[Possible response maybe that only part of it has a side walk. If so, treat this as a YES response. The participant may also respond by saying “no, but the street just around the corner does”. If so, treat this as a NO response. They may ask you to define a side walk. If so, this is a paved path, not a gravel track]

……………………………………………………………………………………………………………….

**HEALTH BEHAVIORS**

**19.** *Are you or anyone else in the home following a weight loss diet?* Yes /No / Don’t know

[A possible response may be that they are supposed to be on a diet, but not good at keeping to it. If so, report YES. They may also say that they have just finished (or are about to start) a diet. If so, report NO]

**If yes** *which family members?* [write members initials and relationship with child below, e.g. participant, reference child, brother, father etc.]

………………………………………………………………………………………………………………………………………………………………………………………………………………………………………………………………………………………………………………………………………………………………………………………………………………………………………………………………

**20.** *Are you or anyone else in the home a member of a gym, YMCA or community center?* Yes /No /Don’t know

**If yes..** *which family members?* [write members initials and relationship with child below, e.g. participant, reference child, brother, father etc.]

[A possible response may be that they are members, but not good at going. If so, report YES. They may also say that their membership has just expired (or is about to start). If so, report NO]

………………………………………………………………………………………………………………………………………………………………………………………………………………………………………………………………………………………………………………………………………………………………………………………………………………………………………………………………

**21.** *Do you or anyone else in the home currently smoke?* Yes /No / Don’t know

**If yes..** *which family members?* [write members initials and relationship with child below, e.g. participant, reference child, brother, father etc.]

[A possible response may be that they suspect other people to smoke, but do not know for sure. If so, report NO for that person. They may also say that they are trying to give up, and are only smoking 1 or 2 cigarettes a day. If so, report YES]

……………………………………………………………………………………………………………………………………………………………………………………………………………………………………………………………………………………………………………………………………………………………………………………………………………………………………………………………....

**22.** *Do you allow smoking in your home?* Yes /No

[A possible response could be that they only allow it in 1 room. If so, report YES]

……………………………………………………………………………………………………………….

**23.** *Do you or anyone else in the home have any medical conditions that impact your diet or physical activities behaviors?* Yes / No

[Provide examples if necessary: diabetes, hypertension, lactose intolerance]

**If yes,** *which family members?* [write initials and relationship with child in the first column of Table 3, e.g. participant, reference child, brother, father etc.].

*For this / each person, please describe:*

1. *what their medical condition is*
2. *whether this affects their diet*
3. *whether it increases or decreases their level of physical activity*

[Begin by asking all questions (a-c) at once and then repeat each question and get a response before moving onto the next question. Fill in responses into Bellow]

Subform_HealthBehavior

| Home ID | Family Member ID | Relationship (Mother, Father, Grandmother, Grandfather, etc) | Medical condition | Diet (Y/N) | PA- increase (I) vs. decrease (D) |
| --- | --- | --- | --- | --- | --- |
|  |  |  |  |  |  |
|  |  |  |  |  |  |
|  |  |  |  |  |  |
|  |  |  |  |  |  |

……………………………………………………………………………………………………………….

**HOME ENVIRONMENT MEASURES**

*Now I’m going to ask you some questions about your home. There may be questions that you are unsure of the answer. It might be that you have to leave the phone and go and look to see what is in your home, otherwise, if you would like to move into your kitchen now, if you have a phone in that room that may help. This is fine. Please answer as honestly as possible and remember that there are no right or wrong answers. You may find some of the questions difficult to answer, but please choose the option that most closely describes your response. The first few questions are going to focus on your family shopping and eating behaviors.*

**H.1.1.** *Do you have any* ***fresh fruit*** *in your home?* Yes / No

**H.1.2.** *Can you tell me what* ***fresh fruits*** *you have in your home?*

[Use the serving size sheet to help you quantify serving sizes]

When the respondent finishes, prompt her by reminding her of places she may have forgotten: *Have you remembered fruits in your refrigerator, in a fruit bowl and in your cupboards?*

Mark the responses for servings in the box below. You will need to prompt participants to let you know how much of each fruit they have.

| **Fresh fruit** |
| --- |
|  |

**H.1.3.** *Would you say that the amount of* ***fresh fruit*** *you currently have in your home is more than usual, less than usual, or about the same?*

More than usual Less than usual The same

**H.1.4.** *Without opening any doors (including doors to your garage, refrigerator or pantry doors) would you be able to see* ***fresh fruit*** *in your home now; displayed out in the open?* Yes / No

[A possible response may be that the fresh fruit is behind a door, but that it is glass and can be seen. If so, report YES. Another response could be that the fresh fruit is out, but that it is stored very high and can only be viewed with a stool. Is so, report NO]

……………………………………………………………………………………………………………….

**H.2.1.** *Do you have any* ***canned or jarred fruits*** *in your home?* Yes / No

**H.2.2.** *If you count a regular size can or jar as being between 14 and 15 ounces, can you tell me how many* ***cans or jars of fruits*** *you have in your home now?*

When the respondent finishes, prompt her by reminding her of places she may have forgotten: *Have you remembered canned fruits in your garage?*

Mark responses in the box below. You will need to prompt participants to let you know how much of each canned/jarred fruit they have.

| **Cans / jars of fruit** |
| --- |
|  |

**H.2.3.** *Would you say that the amount of* ***canned or jarred fruit*** *you currently have in your home is more than usual, less than usual, or about the same?*

More than usual Less than usual The same

……………………………………………………………………………………………………………….

**H.3.1.** *Do you have any* ***dried fruit****, such as raisins, dried apricots, or dates in your home now? This does not include dried fruit that is part of a trail mix* Yes / No

**H.3.2.** *Can you tell me what* ***dried fruit*** *you have in your home?*

[Servings should be recorded by the number of cups]

Mark the responses in the box below. You will need to prompt participants to let you know how much of each dried fruit they have.

| **Dried fruit** |
| --- |
|  |

**H.3.3.** *Would you say that the amount of* ***dried fruit*** *you currently have in your home is more than usual, less than usual, or about the same?*

More than usual Less than usual The same

……………………………………………………………………………………………………………….

**H.41.** Do you have any frozen fruit in your home now? Yes/No

**H.4.2.** *Can you tell me what* ***frozen fruit*** *you have in your home?*

[Servings should be recorded by the number of cups]

You may have to prompt the participant to get an idea of the amount of each frozen fruit: *“roughly how many cups of frozen strawberries do you think there are in your bag?*”

Mark the responses in the box below. You will need to prompt participants to let you know how much of each frozen fruit they have.

| F**rozen fruit** |
| --- |
|  |

**H.4.2.** *Would you say that the amount of* ***frozen fruit*** *you currently have in your home is more than usual, less than usual, or about the same?*

More than usual Less than usual The same

……………………………………………………………………………………………………………….

**H.41.** Do you have any fresh vegetables in your home now? Yes/No

**H.5.1.** *Can you tell me what* ***fresh vegetables*** *you have in your home?*

[Servings should be recorded by number of cups]

When the respondent finishes, prompt her by reminding her of places she may have forgotten: *Have you remembered vegetables in your refrigerator, or in your garage?*

Mark the responses in the box below. Make a mark for each cup in the left column. You will need to prompt participants to let you know how much of each vegetable they have. FRESH VEGETABLES INCLUDE POTATOES AND ONIONS, BUT NOT GARLIC

| **Fresh vegetables** |
| --- |
|  |

**H.5.2.** *Would you say that the amount of* ***fresh vegetables***  *you currently have in your home is more than usual, less than usual, or about the same?*

More than usual Less than usual The same

**H.5.3.** *Do you have any ready to eat* ***fresh vegetables*** *on a shelf in the refrigerator or on the kitchen counter now? These include baby carrots, cherry tomatoes, or vegetables that you have sliced to make them ready to eat.* Yes / No

……………………………………………………………………………………………………………….

**H.6.1.** *Do you have any* ***canned or jarred vegetables*** *in your home?* Yes / No

**H.6.2*.*** *If you count a regular size can as being between 14 and 15 ounces, can you tell me how many* ***cans of vegetables*** *you have in your home now?*

When the respondent finishes, prompt her by reminding her of places she may have forgotten: *Have you remembered canned vegetables in your garage?*

If the participant can not respond in cups, ask her what the weight on the packaging is. Use this to categorize the number of cups after the interview.

Mark responses in the box below. You will need to prompt participants to let you know how much of each canned vegetable they have.

| **Cans of vegetables** |
| --- |
|  |

**H.6.3.** *Would you say that the amount of* ***canned vegetables***  *you currently have in your home is more than usual, less than usual, or about the same?*

More than usual Less than usual The same

……………………………………………………………………………………………………………….

**H.41.** Do you have any frozen vegetables in your home now? Yes/No

**H.7.1.** *Can you tell me what* ***frozen vegetables*** *you have in your home?*

[Servings should be recorded by the number of cups]

You may have to prompt the participant to get an idea of the amount of each frozen vegetable: *“roughly how many handfulls of frozen peas do you think there are in your bag?*”

If the participant can not respond in cups, ask her what the weight on the packaging is. Use this to categorize the number of cups after the interview.

Mark the responses in the box below. You will need to prompt participants to let you know how much of each frozen vegetable they have.

| **Frozen vegetables** |
| --- |
|  |

**H.7.2.** *Would you say that the amount of* ***frozen vegetables***  *you currently have in your home is more than usual, less than usual, or about the same?*

More than usual Less than usual The same

……………………………………………………………………………………………………………….

*Now I’m going to ask you about what snacks you have in your home. Again, please respond as accurately as possible and remember that you are not being judged on your answers.*

**H.2.1.** Do you have any salty snacks in your home? Yes/No

**H.8.1.** *Can you tell me what* ***salty snacks*** *you have in your home? Include snacks like peanuts, chips, tortillas and pretzels, but not popcorn.*

[Use the savory snack sheet to help you identify snacks and serving sizes]

When the respondent finishes, prompt her by reminding her of places she may have forgotten: *Have you remembered snacks in your refrigerator, or in your garage?*

Mark the responses in the box below. You will need to prompt participants to let you know how much of each savory snack they have.

| **Savory snacks** |
| --- |
|  |

**H.8.2.** *Would you say that the amount of* ***savory snacks***  *you currently have in your home is more than usual, less than usual, or about the same?*

More than usual Less than usual The same

**H.8.3.** *Would it be possible for your child to and get any* ***salty snacks*** *on their own, without your help?* Yes / No

[A possible response may be that they do not allow their child to do that without asking, but that they could get if they were permitted. If so, report Yes]

……………………………………………………………………………………………………………….

**H.2.1.** Do you have any sweet snacks in your home? Yes/No

**H.9.1.** *Can you tell me what* ***sweet snacks*** *you have in your home? Include snacks like cookies, ice-cream, Twinkies, muffins and cake. Do not include candy.*

[Use the sweet snack sheet to help you identify snacks and serving sizes]

When the respondent finishes, prompt her by reminding her of places she may have forgotten: *Have you remembered snacks in your refrigerator, or in your garage?*

Mark the responses in the box below. You will need to prompt participants to let you know how much of each sweet snack they have.

| **Sweet snacks** |
| --- |
|  |

**H.9.2.** *Would you say that the amount of* ***sweet snacks***  *you currently have in your home is more than usual, less than usual, or about the same?*

More than usual Less than usual The same

**H.9.3.** *Would it be possible for your child to and get any* ***sweet snacks*** *on their own, without your help?* Yes / No

[A possible response may be that they do not allow their child to do that without asking, but that they could get if they were permitted. If so, report Yes]

……………………………………………………………………………………………………………….

**H.2.1.** Do you have any candy in your home? Yes/No

**H.10.1.** *Can you tell me what* ***candy*** *you have in your home? This includes candy such as hard candy and chocolate bars.*

When the respondent finishes, prompt her by reminding her of places she may have forgotten: *Have you remembered candy in your refrigerator, in your garage, or in a bowl?*

Mark the responses in the box below. You will need to prompt participants to let you know how much of each candy they have.

| **Candy** |
| --- |
|  |

**H.10.2.** *Would you say that the amount of* ***candy*** *you currently have in your home is more than usual, less than usual, or about the same?*

More than usual Less than usual The same

**H.10.3.** *Would it be possible for your child to and get any* ***candy*** *on their own, without your help?* Yes / No

[A possible response may be that they do not allow their child to do that without asking, but that they could get if they were permitted. If so, report Yes]

……………………………………………………………………………………………………………….

**H.2.1.** Do you have any soda in your home? Yes/No

**H.11.1.** *Can you describe to me what* ***soda*** *you have in your home? Tell me how much you have, if any and what size the bottles or cans are. Please do not include diet sodas.*

When the respondent finishes, prompt her by reminding her of places she may have forgotten: *Have you remembered soda in your refrigerator, or in your garage?*

Mark the responses in the box below. Record as much information as possible. For example, the participant response might be “I have 4 cans of sprite – regular size, plus a large 1 liter bottle of coke”. If so, write all of this.

| **Soda** |
| --- |
|  |

**H.11.2.** *Would you say that the amount of* ***soda*** *you currently have in your home is more than usual, less than usual, or about the same?*

More than usual Less than usual The same

**H.11.2.** *Would it be possible for* <child’s name> *to and get* ***soda*** *on their own, without your help?* Yes / No

[A possible response may be that they do not allow their child to do that without asking, but that they could get if they were permitted. If so, report Yes]

……………………………………………………………………………………………………………….

**H.12.1.** *How many days a week does* <child’s name> *eat breakfast at home?*

0 1 2 3 4 5 6 7

[Breakfast includes snacks as well as cereal or toast. Weekly estimates include week days and weekend days. Breakfasts that are prepared at home, but not eaten at home are a “no” response]

**H.12.2.** *How many days a week does* <child’s name> *eat breakfast at school or preschool?*

0 1 2 3 4 5 6 7

[This includes food prepared at home, foods purchased on the way to school and food prepared by the school or pre-school – provided they are eaten at school or preschool]

**H.12.3.** *How many days a week often does* <child’s name> *eat breakfast somewhere else, not including home, school or preschool?*

0 1 2 3 4 5 6 7

[This includes breakfasts that are purchased from a store, garage or fast food restaurant. It does not include breakfast that are eaten at a friends or another family home]

……………………………………………………………………………………………………………….

**H.13.1.** *How many days a week do your family sit at a table to eat dinner together? This includes occasions when it is just* <child’s name> *and yourself.*

0 1 2 3 4 5 6 7

[Give credit to mothers or fathers who find time to sit down and eat with their child at a table, even if it is just a quick affair with just 1 adult present. A possible response might be that they sit down as a family to eat dinner, but it is not a dining table. This is not included]

**H.13.2.** *How often does* <child’s name> *eat* ***breakfast*** *in front of the TV each week?*

0 1 2 3 4 5 6 7

[For items H.13.2 – H.13.5: If child sits at a dining table in the kitchen, but there is a TV on in the room, this is included]

**H.13.3.** *How often does* <child’s name> *eat* ***lunch*** *in front of the TV each week?*

0 1 2 3 4 5 6 7

**H.13.4.** *How often does* <child’s name> *eat* ***dinner*** *in front of the TV each week?*

0 1 2 3 4 5 6 7

**H.13.5.** *How often does* <child’s name> *eat* ***snacks*** *in front of the TV each week?*

0 1 2 3 4 5 6 7

**H.13.6.** *How often does* <child’s name> *eat dinner away from home each week?*

0 1 2 3 4 5 6 7

[This is only for **dinner**, and does not include meals eaten during the day at school. Do not include dinners eaten at another family home (e.g. if parents are separated)]

**H.13.7.** *From the following options, please tell me where are most meals eaten in your home:*

*a) At the dinning table; b) On the sofa or couch; c) At the coffee table; or d) somewhere else*

[A possible response might be that it is varied, or dependent on the meal. Ask participants to take into account meals not eaten at home, and meals eaten during the weekend so that they can best estimate which place food is most commonly eaten]

……………………………………………………………………………………………………………….

**H.14.1.** *Do you ask* <child’s name> *to eat everything on their plate at dinner; a) all of the time; b) most of the time; c) some of the time; d) rarely; or e) never?*

[This includes participants who make their child eat a majority of foods on their plate. It does not include participants who ask their child to eat certain foods]

**H.14.2.** *Do you restrict dessert if* <child’s name> *does not eat the food on their plate at dinner: a) all of the time; b) most of the time; c) some of the time; d) rarely; or e) never?*

[For items H.14.2 – H.14.4: Include if restriction occurs where: all foods must be finished; a majority of foods must be finished; and certain foods must be finished]

**H.14.3.** *Do you reward* <child’s name> *with desserts, snacks or candy if they finish foods from their plate at dinner: a) all of the time; b) most of the time; c) some of the time; d) rarely; or e) never?*

**H.14.4.** *Do you allow* <child’s name> *to have seconds if they finish foods from their plate at dinner; a) all of the time; b) most of the time; c) some of the time; d) rarely; or e) never?*

**H.14.5.** *Do you generally allow* <child’s name> *to eat only at set meal times: a) all of the time; b) most of the time; c) some of the time; d) rarely; or e) never?*

**H.14.6.** *Do you allow* <child’s name> *to serve themselves at dinner: a) all of the time; b) most of the time; c) some of the time; d) rarely; or e) never?*

[Include if the child is allowed to serve themselves some foods, with the help of others]

**H.14.7.** *Do you allow* <child’s name> *to help themselves to snacks, including salty and sweet snacks, or candy when they are at home: a) all of the time; b) most of the time; c) some of the time; d) rarely; e) never?*

[This does not include if the child has to ask for permission first. Only include if the child is free to help themselves without asking permission]

Refer back to the snack sheets if the participant asks you to describe or clarify snacks

**H. 14.8.** *Would you say that you serve the “same amount”, “more” or “less” dinner to* <child’s name> *compared to what you serve yourself?*

same amount more less

[Response should be for a majority of time. If the child eats completely different meals from the participant, ask the participant to consider serving sizes, or portion sizes, or how big the meal looks on the plate. You could also ask the participant to imagine eating the same foods and whether they think they would eat the same amount, more or less of it]

**H.14.9.** *Do you ever avoid eating savory or sweet snacks, candy or soda in front of* <child’s name>*: a) all of the time; b) most of the time; c) some of the time; d) rarely; e) never?*

[Participant may say that they have to eat in front of the child, because they are always together, but that they try to make it discrete. This response counts as avoiding eating in front of the child]

Refer back to the snack sheets if the participant asks you to describe or clarify snacks

**H.14.10.** *When eating in front of* <child’s name>*, do you try to eat healthy: a) all of the time; b) most of the time; c) some of the time; d) rarely; e) never?*

[A possible response may be, “what do we consider ‘healthy’? This is a subjective answer. It includes what the participant thinks is ‘healthy’]

……………………………………………………………………………………………………………….

**H.15.1.** *Would you say that you have adequate counter space to prepare food in your kitchen? Yes / No*

[Participant may say that they would like more, but that what they have is adequate. If so, report Yes. Another response may be that they have space, but it is covered with equipment / jars / junk. If so, report No]

**H.15.2.** *Which of the following options most closely resembles the way you shop for food?*

You will probably have to repeat this list a number of times before the participant is able to respond.

1. *Monthly big trip*
2. *Every other week, big trip, no small trips*
3. *Every other week, big trip, few small trips*
4. *Weekly, big trip, no small trips*
5. *Weekly, big trip, few small trips*
6. *No big trip, all small as needed*

**H.15.3.** *Does* <child’s name> *help you shop for groceries at the store? For example, you may get them to pick their own foods, or give them their own grocery list. Please consider this for one of the following options: a) all of the time; b) most of the time; c) some of the time; d) rarely; e) never?*

How many days has it been since you last shopped for food?

Was the last shop big or small? Big/Small

[Participants may respond before you get a chance to read them the options. Let them finish and then say, “*ok, can you tell me whether this happens a) all of the time* etc*..”]*

……………………………………………………………………………………………………………..

*OK, that was the last question about food and shopping. The next few questions will ask you about space and equipment in your home.*

**H.16.1***. Do you have yard or open play space that* <child’s name> *can play in?* Yes / No

[This includes shared yard space for people living in apartments, but does not include park space, even if it is very close to the home]

**H.16.2.** *Would you say that your yard space is small, medium or large?*

small medium large

[This is a subjective question. Try to get the participant to answer what they feel the size of their yard is]

**If no,** go straight to H.16.5 and skip H.16.8

**If yes,** go to H.16.2

**H.16.3.** *Do you share your yard with other households?* Yes / No

[This does not include park land]

**H.16.4.** *Do you have any usable play equipment such as swings, slides, climbing or ladders in your yard?* Yes / No

[Usable means that it is ready to use. For example, swings are well grounded and have chairs]

**H.16.5.** *Does* <child’s name> *have a useable tricycle, bike, scooter or wheeled toy?* Yes / No

[Usable means that it is ready to use. For example, bikes have tires that are pumped up and chains that are not broken]

*The next couple of questions are about active play. By “active play” we mean when* <child’s name> *is physically moving during playing, like running, jumping, peddling, or climbing.*

**H.16.6.** *To what extent would you agree that* <child’s name> *has adequate room to play actively inside the home: a) strongly disagree; b) somewhat disagree; c) somewhat agree; or d) strongly agree?*

[A possible response maybe that there is space in some rooms, but not in others. Get the participant to consider this with their response. For example, if there is only space in 1 room, the answer might be c) somewhat agree]

**H.16.7.** *Would you say that you restrict active play indoors: a) all of the time; b) most of the time; c) some of the time; d) rarely; e) never?*

[Explanations for items H.16.7 – H.16.9 are irrelevant. It might be that participants restrict play most of the time because they do not feel that it is safe. This response should remain as b) most of the time]

**!!!!H.16.8.** *Would you say that you restrict outdoor play in your yard: a) all of the time; b) most of the time; c) some of the time; d) rarely; e) never?*

[Potential response may be that the child is only allowed to play outside if an adult is present. If play is never restricted within that parameter, circle “e) never”]

**H.16.9.** *Would you say that you restrict outdoor play in your immediate neighborhood: a) all of the time; b) most of the time; c) some of the time; d) rarely; e) never?*

……………………………………………………………………………………………………………..

**H.17.1.** *During the past month, other than your regular job, did you participate in any physical activities or exercises such as running, calisthenics, golf, gardening or walking for exercise?* Yes / No

**If No,** go straight to H.18.1

**H.17.2.** *From the following responses, how often would you say that you are active in the presence of your child: a) always; b) most of the time; c) some of the time; d) rarely, e) never?*

[This includes if the participant takes the child to the gym with them, even if the child is in a crèche where they are not able to actually see them exercise]

……………………………………………………………………………………………………………..

*Ok, now we’re on to the last set of questions, which will be about TV and media in your home*.

**H.18.1.** *How many working TV’s do you have in your home?*

0 1 2 3 4 >4

[Include TV’s that are temporarily broken if there is a plan to get them fixed]

**H.18.2.** *Do you have cable or satellite?* Yes / No

**If No,** go straight to H. 18.4

**H.18.3.** *Can you estimate the number of channels you have available to you?*

0 1-4 5-10 11-30 31-49 50-100 >100

[This may be a difficult question to answer. If the participant is having problems, prompt them with questions related to the categories above. For example: “*would you say that you have more than 100 channels?”*]

**H.18.4.** *How many working VCR or DVD players do you have in your home?*

0 1 2 3 4 >4

[Include VCR’s or DVD players that are temporarily broken if there is a plan to get them fixed. Also include DVD’s within computers if they are used to watch movies on]

**H.18.5.** *How many DVDs or Video tapes do you have in your homes that are specifically for your child to watch?*

0 1-4 5-10 10-25 >25

[DVD’s or tapes that are shared by the whole family are not included. Only include those which are exclusively for the child. Do not include ones that are exclusive for other children, unless the target child also watches them]

**H.18.9.** *Does* <child’s name> *have a working TV in their bedroom?* Yes / No

[Include TV’s if it is a shared bedroom and the TV belongs to another child]

**H.18.10.** *Do you have any working TV’s that are viewable from your dining area (or the food where most meals are eaten)?* Yes / No

[Include even if the participant says, “yes, but it is never switched on during meal time”]

……………………………………………………………………………………………………………..

**H. 19.1.** *How many working computers or laptops do you have in your home?*

0 1 2 3 4 >4

[Include computers or laptops that are temporarily broken if there is a plan to get them fixed]

**H.19.2.** *Does* <child’s name> *have a computer or laptop in their bedroom?* Yes / No

[Include computers, if it is a shared bedroom and the TV belongs to another child]

……………………………………………………………………………………………………………..

**H.20.1.** *How many working games consoles, such as Play Station or X-Box, do you have in your home?*

0 1 2 3 4 >4

[Include game consoles that are temporarily broken if there is a plan to get them fixed]

**H.20.2.** *Does* <child’s name> *have a games console in their bedroom?* Yes/No

[Include games consoles that are temporarily broken if there is a plan to get them fixed]

……………………………………………………………………………………………………………..

**H.21.1.** *From the following options, how often would you say that you restrict the amount of time* <child’s name> *spends watching TV: a) always; b) most of the time; c) some of the time; d) rarely; e) never?*

[For items H.21.1 – H.21.3; restriction means that they do not allow their child to watch TV / use the computer / play games consoles, for what ever reason. This includes evenings and weekends. Participants may say that they only restrict TV time in the morning. If so, ask them if they would therefore respond as some of the time? Participants who restrict during the week, but not at weekends should respond as most of the time. Those that restrict only certain TV programs should report rarely]

**H.21.2.** *From the following options, how often would you say that you restrict the amount of time* <child’s name> *spends using a computer or laptop: a) always; b) most of the time; c) some of the time; d) rarely; e) never?*

**H.21.3.** *From the following options, how often would you say that you restrict the amount of time* <child’s name> *spends playing games on the games console: a) always; b) most of the time; c) some of the time; d) rarely; e) never?*

**H.21.4.** *From the following options, how often would you say that you reward good behavior with extra TV time: a) always; b) most of the time; c) some of the time; d) rarely; e) never?*

[For items H.21.3 – H.21.5; good behavior is subjective and depends on what the participant considers to be good behavior. It may be that they have been quiet or that they have eaten their vegetables]

**H.21.5.** *From the following options, how often would you say that you reward good behavior with extra computer time: a) always; b) most of the time; c) some of the time; d) rarely; e) never?*

**H.21.6.** *From the following options, how often would you say that you reward good behavior with extra game/game console time: a) always; b) most of the time; c) some of the time; d) rarely; e) never?*

*That’s the end of the interview now. Thank you very much for your time. We now need to arrange a convenient time for us to come and visit you in your home.*

*On the availability sheet, you suggested that* [list days and times] *would be the best time to come to your home. Can we schedule a time to suit you within the next week?*

Schedule an appointment

*Thank you again, and goodbye*

End time:
